# Transcriptional downregulation of *agr* expression in *Staphylococcus aureus* during growth in human serum can be overcome by constitutively active mutant forms of the sensor kinase AgrC

**DOI:** 10.1111/1574-6968.12309

**Published:** 2013-11-18

**Authors:** Ellen H James, Andrew M Edwards, Sivaramesh Wigneshweraraj

**Affiliations:** Faculty of Medicine, MRC Centre for Molecular Bacteriology and Infection, Imperial College LondonLondon, UK

**Keywords:** transcription regulation, GFP transcriptional reporters, two-component systems

## Abstract

The temporal and cell density-dependent regulation of expression of virtually all the *Staphylococcus aureus* virulon is under the control of the *agr* (accessory gene regulatory) operon. The expression of the *agr* operon is subject to transcriptional regulation by the AgrA/C two-component response regulator/sensor kinase pair. During bacteraemia, a frequent syndrome caused by methicillin-resistant *S. aureus* (MRSA), the transcriptional downregulation of *agr* expression has been attributed to the sequestration of the quorum-signalling molecule auto-inducing peptide (AIP) by the human serum component apolipoprotein B as part of an innate immune response to infection. However, it is not known whether transcriptional downregulation of *agr* expression during growth in human serum is additionally subjected to regulation by transcription regulatory proteins that either directly or indirectly affect transcription from the *agr* operon promoters. Here, using chromosomal fluorescence reporters of *agr* expression in *S. aureus,* we show that the transcriptional downregulation of *agr* expression in human serum can be overcome using constitutive active mutant forms of AgrC. Therefore, it seems that the sequestration of the AIP is likely to be the only mechanism by which the host innate immune response limits *agr* expression at the transcriptional level to maintain the host–pathogen balance towards a noninvasive outcome.

## Introduction

The cell density-dependent modulation of gene expression often manifests itself at the transcriptional level and involves a two-component signal transduction system. The cell density or quorum-signalling molecule, the autoinducer, is sensed by the receptor kinase, which modulates the activity of the response regulator, leading to alterations in the transcription patterns of target genes (Stock *et al*., 2000; West & Stock, 2001) Gram-positive bacteria use secreted peptides as autoinducers (Novick & Geisinger, 2008). The Gram-positive bacterial pathogen *Staphylococcus aureus* causes a wide variety of life-threatening invasive infections in humans. The pathogenic success of *S. aureus* can be attributed to the diverse array of virulence factors, involving a large number of cell-surface bound proteins (e.g. adhesins, fibrinogen/fibronectin binding proteins) that are expressed during colonisation of the host, and secreted proteins (e.g. haemolysins, proteases, lipases) that are required for acute infections (Dunman *et al*., 2001; Cheung *et al*., 2011).

An auto-inducing quorum-sensing system encoded within the *agr* operon in part coordinates the phenotypic change in *S. aureus* during infection from adhesive and colonising to tissue damaging and invasive (Novick & Geisinger, 2008). Therefore, the coordinated regulation of *agr* operon expression is an important criterion for the pathogenic success of *S. aureus,* at least during the acute stage of infection (Cheung *et al*., 2011). However, it should be noted that the role of *agr* after infection is established is less clear and mutations that inactivate *agr* are sometimes found in clinical isolates (Traber *et al*., 2008; Shopsin *et al*., 2010).

**Figure 1:**
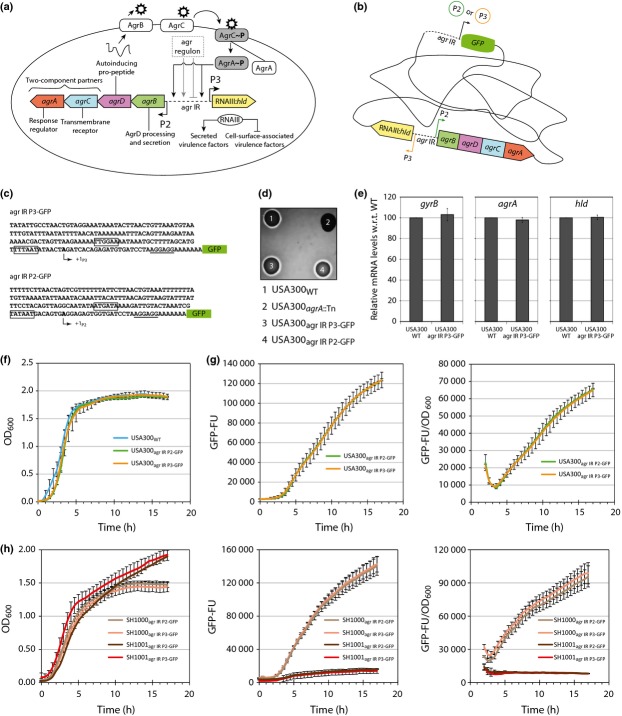
(a) Schematic representation of the *agr* operon organisation and regulation in *Staphylococcus aureus*. (b) Schematic representation showing single-site chromosomal integration of GFP transcriptional fusion reporters for P2 and P3. (c) Sequence of P2 and P3 promoter regions used to generate the *gfp* transcriptional fusions for P2 and P3 (the consensus −10 and −35 sites are outlined, the transcription start sites are shown with arrows and the RBS is underlined. (d) Sheep blood agar haemolysis assay with *S. aureus* USA300_WT_ (1), USA300_*agrA*::Tn_ [*agrA* transposon mutant from NARSA library (Fey *et al*., 2013)] (2), USA300_agr IR P3-GFP_ (3) and USA300_agr IR P2-GFP_ (4). (e) Graphs showing relative *hld*, *agrA* and *gyrB* mRNA levels in 16 h cultures of *S. aureus* USA300_WT_ and USA300_agr IR_ _P3-GFP_ as determined by Taqman qRT-PCR. Values are shown for each gene with respect to USA300_WT_ levels. (f) Graph showing growth curves (OD_600_) of *S. aureus* USA300_WT_, USA300_agr IR P2-GFP_ and USA300_agr IR P3-GFP_ strains grown in TSB. (g) Graphs showing GFP expression [as GFP fluorescence units (GFP-FU)] and GFP-FU as a function of growth (OD_600_) over time for *S. aureus* USA300_agr IR P2-GFP_ and USA300_agr IR P3-GFP_ strains grown in TSB. (h) As in (g) but with *S. aureus* SH1000_agr IR_ _P2-GFP_, SH1000_agr IR P3-GFP_, SH1001_agr IR_ _P2-GFP_ and SH1001_agr IR_ _P3-GFP_ strains grown in TSB. Data for (d–h) were obtained from three biological replicates

The *agr* operon, conserved in all *Staphylococcus* isolates examined thus far (Novick & Geisinger, 2008; Wuster & Babu, 2008), is expressed from divergent promoters, P2 and P3; where P2 encodes a quorum-sensing system and P3 encodes a pleiotropic effector of the virulon (Fig.1a; Koenig *et al*., 2004). Consistent with the central role of the *agr* operon in regulating the expression of the *S. aureus* virulon, a vast array of transcription regulatory proteins either directly or indirectly control transcription from P2 and P3 (Novick & Geisinger, 2008; Reyes *et al*., 2011). In addition, AgrA is also subjected to post-translational modification under specific growth conditions, and *agrA* mRNA is also post-transcriptionally regulated in some *S. aureus* strains (Sun *et al*., 2012; Kaito *et al*., 2013).

Recent evidence indicates that elements of host innate immunity regulate the *agr* operon-mediated phenotypic changes in *S. aureus* during infection, from adhesive and colonising to tissue damaging and invasive, thereby contributing to the maintenance of the host–pathogen balance in favour of a noninvasive outcome (Rothfork *et al*., 2004; Peterson *et al*., 2008; Malachowa *et al*., 2011; Hall *et al*., 2013). The sequestration of AIP by apolipoprotein B (which is present in blood that extravasates to site of acute infection) represents a primary host innate immune mechanism to downregulate *agr* operon expression at the transcriptional level to limit invasive infections caused by *S. aureus* (Peterson *et al*., 2008; Hall *et al*., 2013). The sequestration of AIP by apolipoprotein B renders the AIP unavailable to interact with its membrane-bound receptor AgrC and thereby compromises the efficiency by which AgrC activates its cognate response regulator AgrA. This subsequently leads to downregulation of transcription from P2 and P3 by AgrA (Peterson *et al*., 2008; Hall *et al*., 2013). AIP can also be inactivated by oxidants, for example NADPH (Rothfork *et al*., 2004). However, it is not known whether downregulation of *agr* expression is limited to the sequestration of AIP by apolipoprotein B or whether it additionally involves regulation of P2 and P3 activity by the other transcription regulatory proteins that affect transcription from P2 and P3 or through post-translational regulation rendering AgrA unavailable or inactive for the activation of transcription from P2 and P3. Here, we present results from experiments in which we have addressed this issue in the context of the community-associated methicillin-resistant *S. aureus* (CA-MRSA) strain USA300 LAC* (hereafter referred to as USA300). The USA300 lineage is the most frequent cause of CA-MRSA bacteraemia in the United States and causes the most invasive forms of infection (Klevens *et al*., 2007).

## Materials and methods

### Bacterial strains, plasmids and DNA manipulation

The bacterial strains and plasmids used in this study are listed in Table1. *Escherichia coli* and *S. aureus* were grown in Luria broth (LB) and tryptic soy broth (TSB), respectively. The sequences of primers used for DNA manipulation and cloning are listed in Supporting Information, Table S1. Human serum (derived from male AB plasma, sterile-filtered) was purchased from Sigma. Further details of reagents, bacterial growth conditions and DNA manipulation techniques can be found in the Supporting Information, Data S1 (see also Fig.1b and 1c for information regarding construction of transcription reporters).

**Table 1:** Bacterial strains and plasmids used

Strain/plasmid	Genotype/characteristics^*^	Source/reference
Strains		
* E. coli*		
XL1-blue	Efficient cloning strain	Stratagene
*S. aureus*		
RN4220	NCTC8325-4, restriction mutant	Peng *et al*. (1988)
SH1000	Functional *rsbU* derivative of 8325-4 *rsbU*	Horsburgh *et al*. (2002)
SH1001	SH1000 *agr*::*tet*	Horsburgh *et al*. (2002)
NE1532	USA300 LAC *agrA* transposon mutant, here called USA300_*agrA*::Tn_	NARSA library Fey *et al*. (2013)
USA300 LAC^*^	Erm-sensitive CA-MRSA LAC^*^ strain; hereafter called USA300_WT_	Boles *et al*. (2010)
SH1000_agr IR_ _P3-GFP_	pCL55_agr IR_ _P3-GFP_ integrated into SH1000	This study
SH1000_agr IR_ _P2-GFP_	pCL55_agr IR_ _P2-GFP_ integrated into SH1000	This study
SH1001_agr IR_ _P3-GFP_	pCL55_agr IR_ _P3-GFP_ integrated into SH1001	This study
SH1001_agr IR_ _P2-GFP_	pCL55_agr IR_ _P2-GFP_ integrated into SH1001	This study
USA300_agr IR_ _P3-GFP_	pCL55_agr IR_ _P3-GFP_ integrated into USA300_WT_	This study
USA300_agr IR_ _P2-GFP_	pCL55_agr IR_ _P2-GFP_ integrated into USA300_WT_	This study
Plasmids		
pCL55	Single-site integration vector for *S. aureus*. Amp^R^ in *E. coli*, Cm^R^ in *S. aureus*	Lee *et al*. (1991)
pCN34	Shuttle vector for Gram-positive bacteria. Amp^R^ in *E. coli*, Kan^R^ in *S. aureus*	Charpentier *et al*. (2004)
pCL55_agr IR_ _P3-GFP_	pCL55 carrying P3GFP	This study
pCL55_agr IR_ _P2-GFP_	pCL55 carrying P2GFP	This study
pCN34_*agrC* WT_	pCN34 carrying P2_*agrC* WT_	This study
pCN34_*agrC* M234L_	pCN34 carrying P2_*agrC* M234L_	This study
pCN34_*agrC* R238H_	pCN34 carrying P2_*agrC* R238H_	This study
pCN34_*agrC* Q305H_	pCN34 carrying P2_*agrC* Q305H_	This study

* Amp, ampicillin; Cm, chloramphenicol; tet, tetracycline; Kan, kanamycin; Erm, erythromycin.

### Blood agar haemolysis assay

Strains were grown for 16 h in TSB culture, then 5 μL aliquots were subcultured onto tryptic soy agar containing 5% sheep's blood and left to grow for 16 h at 37 °C.

### Real-time quantitative reverse transcription PCR (qRT-PCR)

Details of RNA extraction and cDNA synthesis can be found in the Data S1. qRT-PCR was performed using primers and Taqman probes corresponding to *hld* (delta toxin), *agrA* and *gyrB* (gyrase B) with QPCR core kit, no ROX (Eurogentec) according to the manufacturer's instructions. Reactions were performed in an ABI PRISM 7700.

### Bacterial growth and GFP expression assays

These were conducted as described previously for *E. coli* (Shadrin *et al*., 2012). Simultaneous growth (OD_600_) and GFP fluorescence measurements (with excitation and emission filters of 485 and 520 nm respectively) were performed in 96-well black microtitre plates with clear bottoms (Corning) in a POLARstar Omega multiwell plate reader (BMG Labtech). Three biological replicates (each defined as a single colony) were performed for each growth curve. Further details can be found in the Data S1.

### Western blotting to determine intracellular levels of AgrA

Details of the experimental set-up and sample preparation can be found in the Data S1 (see also Fig.3). Western blotting was performed using polyclonal antibodies against AgrA (raised in rabbits against untagged recombinant AgrA by Eurogentec; used at 1 : 500 dilution) and anti-rabbit–horseradish peroxidase-conjugated antibodies (Dakocytomation; used at 1 : 10 000 dilution) as primary and secondary antibodies, respectively, following standard laboratory protocols.

## Results

### Development of a near real-time fluorescence-based system to monitor transcription from *agr* operon promoters P2 and P3

To study how transcription from P2 and P3 is affected during growth in serum, two *S. aureus* USA300 reporter strains were created by ‘ectopically’ placing transcriptional fusions of P2 or P3 to GFP at the *geh* locus on the *S. aureus* chromosome using the integration plasmid pCL55 whilst leaving the native *agr* operon intact (Fig.1b; Lee *et al*., 1991). Each transcriptional fusion construct consisted of either the P2 or the P3 promoter sequence and the *agr* IR sequence up to, but not including, the consensus −35 promoter element of the divergent promoter upstream of the gene encoding GFP with an optimal RBS sequence (Fig.1c).

**Figure 2:**
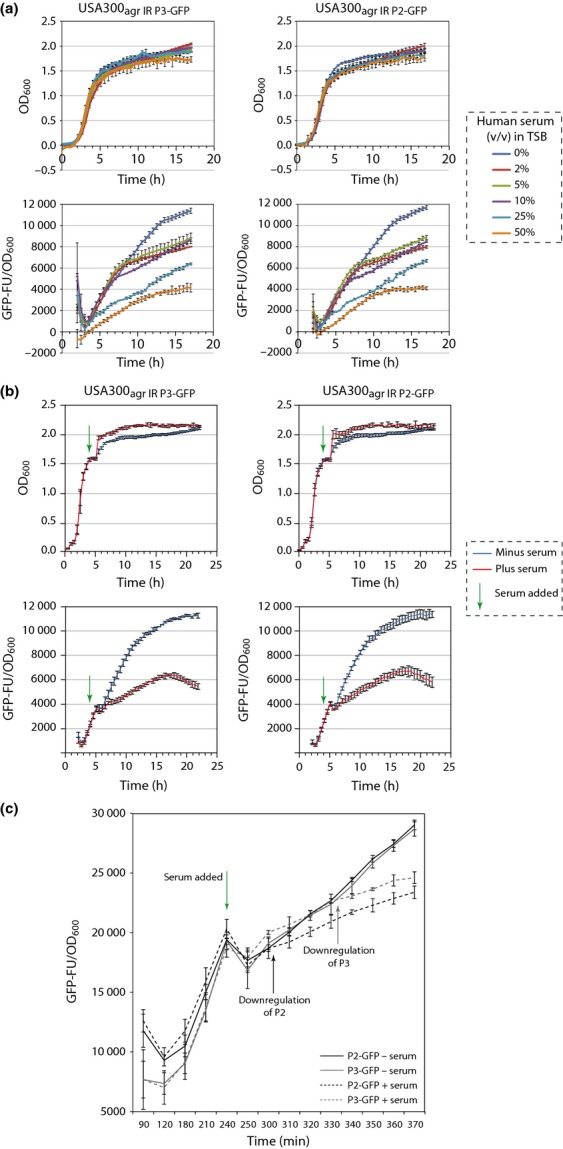
(a) Graphs showing GFP expression [as GFP fluorescence units (GFP-FU)] as a function of growth (OD_600_) over time for *Staphylococcus aureus* USA300_agr_ _IR__P2-__GFP_ and USA300_agr_ _IR__P3-__GFP_ strains grown in TSB media containing 0–50% (v/v) human serum. (b) Graph showing GFP expression (as GFP fluorescence units (GFP-FU)) as a function of growth (OD_600_) over time for USA300_agr_ _IR__P2-__GFP_ and USA300_agr_ _IR__P3-__GFP_ strains grown in TSB media upon addition of 25% (v/v) human serum to late-exponentially growing cells (indicated with a green arrow). (c) As in (b), however, readings for OD_600_ and GFP expression values were taken every 30 min before the addition of human serum and every 10 min thereafter. Data for graphs (a–c) were obtained from three biological replicates.

As we wished to conduct downstream assays with the USA300 reporter strains in the absence of antibiotic chloramphenicol (pCL55 carries the *cat* gene that confers resistance to chloramphenicol), we initially determined the stability of the integrated reporter constructs: after 24 h growth in TSB (without chloramphenicol), bacteria containing P2 and P3 reporter constructs were cultured on TSA in the absence of antibiotic and 28 colonies from each plate (*n* = 3) were examined for chloramphenicol resistance on TSA containing chloramphenicol. In each colony, chloramphenicol resistance was maintained and all colonies emitted green fluorescence when exposed to blue light (Supporting Information, Fig. S1). We therefore conclude that the reporter constructs are extremely stable under the experimental conditions used. We next tested whether the presence of an additional copy of the *agr* IR sequence in the *S. aureus* USA300_agr IR P2-GFP_ and USA300_agr IR P3-GFP_ reporter strains affected the *agr* operon expression (e.g. by titrating away regulatory interactions at the native *agr* IR) and the growth of *S. aureus* USA300 strain in any way: as *agr* dysfunction is associated with β-haemolytic activity, we used a blood agar plate haemolysis assay to establish that the *S. aureus* USA300_agr IR P2-GFP_ and USA300_agr IR P3-GFP_ reporter strains are not compromised for β-haemolysis (Fig.1d). Further, we also isolated total RNA from *S. aureus* USA300_WT_ and reporter strains USA300_agr IR P2-GFP_ and USA300_agr IR P3-GFP_ and determined the mRNA levels of *agrA* (reports native P2 transcription) and *hld* (reports native P3 transcription) relative to that of *gyrB* (reports transcription of the constitutively expressed gene encoding gyrase) by qRT-PCR. As shown in Fig.1e (and data not shown), and consistent with the results from Fig.1d, no obvious differences in the levels of *agrA* and *hld* mRNA were detected between *S. aureus* USA300_WT_ and reporter USA300_agr IR P3-GFP_ strains. In addition, the growth rate of *S. aureus* USA300_WT_, USA300_agr IR P2-GFP_ and USA300_agr IR P3-GFP_ strains did not significantly differ in TSB (Fig.1f). Having established that the presence of an additional copy of the *agr* IR sequence with either the P2 or P3 did not detectably affect native *agr* expression, *agr*-mediated downstream effects and the growth characteristics of *S. aureus* USA300_WT_, we measured the GFP expression as a function of growth in *S. aureus* USA300_agr IR P2-GFP_ and USA300_agr IR P3-GFP_ reporter strains. The GFP signal, originating from the ectopically placed P2 and P3, increases concomitantly with the increase in cell density in both reporter strains grown in TSB (Fig.1g). The rate of GFP expression from P2 and P3 is very similar, thus indicating that transcription from the ectopically placed P2 and P3 occurs at a similar rate under our experimental conditions. However, in contrast, a previous study using plasmid-based GFP reporters of P2 and P3 activity in *S. aureus* strain SH1000 (Reyes *et al*., 2011) showed that GFP expressed from P3 accumulates at a much faster rate than GFP expressed from P2, thus implying that P3 is a stronger promoter than P2. To rule out any strain-specific factors and/or unregulated transcription from the P2 and P3 transcriptional fusion constructs at the *geh* locus accounting for similar activity of P2 and P3 under our experimental conditions, we placed transcriptional fusions of P2 or P3 to GFP at the *geh* locus on the chromosome of *S. aureus* strain SH1000 and the *agr* operon-deficient mutant strain SH1001 (Horsburgh *et al*., 2002; Table1). As with the USA300 reporter strains, the GFP signal originating from P2 and P3 increased concomitantly with an increase in cell density and at a similar in the SH1000 reporter strain (Fig.1h). However, as expected, no transcription form P2 and P3 is detected in the SH1001 reporter strain. Therefore, even though we are unable to differentiate the specific activities of P2 and P3 during growth in the context of our chromosomal reporter strains and under our experimental conditions (but see later, Fig.2c), we are confident that the GFP signal originating from the ectopically placed P2 and P3 faithfully mirrors the regulatory events that occur at the native *agr* operon.

### Downregulation of transcription from *agr* operon promoters in response to human serum

To examine the effect of human serum on P2 and P3 transcription in the *S. aureus* USA300_agr IR P2-GFP_ and USA300_agr IR P3-GFP_ reporter strains, bacteria were grown in TSB supplemented with a range of concentrations of human serum [5–50% (v/v) final concentration] and measured GFP expression as a function of growth. As expected, the reduction in GFP expression positively correlated with the increase in human serum concentration in the growth medium (Fig.2a). As 25% (v/v) human serum did not detectably affect growth yet still caused a significant downregulation of transcription from P2 and P3 activity (Fig.2a), we used this concentration of human serum in further experiments.

To test whether the transcriptional downregulation P2 and P3 transcription can be ‘induced’ by human serum, bacteria were grown to late-exponential phase in TSB to allow GFP expression (indicating transcription from P2 and P3) to reach a approximately one-fifth of the maximal level, then added either 25% (v/v) human serum or 25% (v/v) fresh TSB media to the cultures. As shown in Fig.2b, whereas the growth rate of the *S. aureus* USA300_agr IR P2-GFP_ and USA300_agr IR P3-GFP_ reporter strains remains unaffected, GFP expression, indicating transcription from P2 and P3, became detectably reduced. Having established that the addition of human serum to exponentially growing *S. aureus* resulted in downregulation of *agr* operon expression, the time difference between the responses of downregulation of P2 and P3 transcription to human serum was investigated. As P3 transcription is dependent upon AgrA and *agrA* is transcribed from the P2 promoter, we expected that the downregulation of P3 activity would follow downregulation of P2 activity. In agreement with this, the results in Fig.2c show that the transcriptional activity of P2 drops after *c*. 20 min, whereas P3 drops *c*. 50 min after addition of human serum to the cultures. Overall, the results in Figs1 and 2 are consistent with the view that sequestration of AIP by apolipoprotein B in human serum leads to the downregulation of transcription from *agr* operon promoters P2 and P3 and unambiguously demonstrate that *S. aureus* USA300_agr IR P2-GFP_ and USA300_agr IR P3-GFP_ strains report transcription regulatory events at the native P2 and P3 promoters of the *agr* operon in a sensitive and faithful manner (Peterson *et al*., 2008; Hall *et al*., 2013).

### Intracellular levels of AgrA remain unchanged upon addition of human serum to exponentially growing *S. aureus* cells

**Figure 3:**
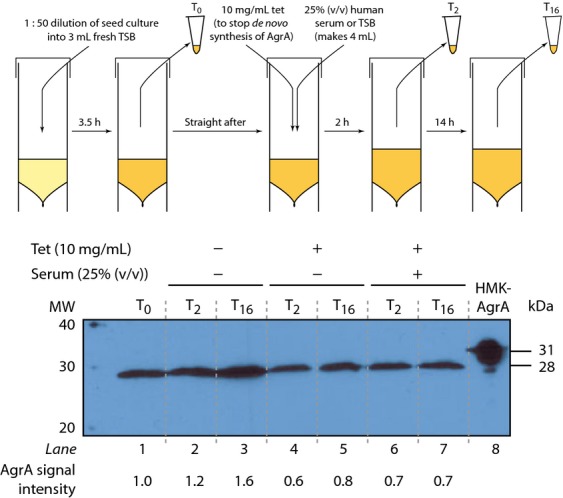
Western blot indicating intracellular AgrA levels in *Staphylococcus aureus* strain USA300_WT_ during growth in TSB medium in the absence and presence of human serum and/or tetracycline. A schematic representation of the experimental steps is shown on the top of the blot (see text for details). Samples of whole-cell extracts were prepared for the analysis immediately before and 2 and 16 h after the addition of human serum (T_0_, T_2_ and T_16_ respectively). Lane 8 contains purified recombinant HMK-tagged AgrA as a positive control marker (Reynolds & Wigneshweraraj, 2011). Data shown are representative from three independent experiments (biological repeats). AgrA signal intensity values were calculated by densitometry using a Typhoon FLA 7000 and indicated relative to the signal intensity at T_0_.

As AgrA can be subjected to post-transcriptional and post-translational regulation (Sun *et al*., 2012; Kaito *et al*., 2013), we considered the possibility that AgrA is subjected to regulation that renders it unavailable or inactive for the activation of transcription from P2 and P3 during growth in human serum. We therefore compared intracellular AgrA levels in *S. aureus* USA300_WT_ cells grown in the presence and absence of human serum. The bacterial cells were grown to late-exponential phase in TSB, and downregulation of *agr* operon expression was induced by adding human serum. At the same time, 10 μg mL^−1^ of tetracycline was added to the cells to prevent *de novo* translation of AgrA (see schematic Fig.3). As the results in Fig.2c indicated that downregulation of transcription from P3 occurred after *c*. 50 min after the addition of human serum, cell samples were taken for analysis by Western blotting using polyclonal anti-AgrA antibodies immediately before (T_0_) and 2 (T_2_) and 16 (T_16_) hours after the addition of human serum. We reasoned that if the addition of human serum to exponentially growing *S. aureus* USA300_WT_ made AgrA unavailable (e.g. by reduction of its half-life), then the intracellular levels of AgrA should be detectably lower in cells to which human serum and tetracycline are added than in cells to which only tetracycline is added. Initially, we confirmed that our anti-AgrA antibody specifically recognises AgrA using whole-cell extracts prepared from *S. aureus* SH1000 strain and the AgrA-deficient mutant *S. aureus* SH1001 (Horsburgh *et al*., 2002; Fig. S2). As expected, in the absence of tetracycline and human serum, AgrA is detected in the T_0_ sample obtained from late-exponentially growing cells (Fig.3, lane 1); a moderate increase in AgrA levels is seen in the T_2_ and T_16_ samples as AgrA is already expressed as maximal level in late-exponentially growing cells at T_0_ (Fig.3, compare lane 1 with lanes 2 and 3). Also as expected, the addition of tetracycline leads to no significant increase in AgrA levels in the T2 and T16 sample compared with the T0 sample (Fig.3, compare lane 1 with lanes 4 and 5). Similarly, the addition of human serum to exponentially growing *S. aureus* USA300_WT_ cells did not detectably reduce intracellular levels of AgrA in the T2 and T16 sample compared with the T0 sample (compare lane 4 and 5 with lanes 6 and 7). The reduction in AgrA levels observed between T0 and tetracycline-containing samples at T2 and T16 likely reflects the half-life of AgrA in the absence of *de novo* protein synthesis. In summary, we conclude that the half-life of AgrA is not detectably affected by human serum.

### Constitutively active mutants of AgrC overcome downregulation of *agr* expression in human serum

**Figure 4:**
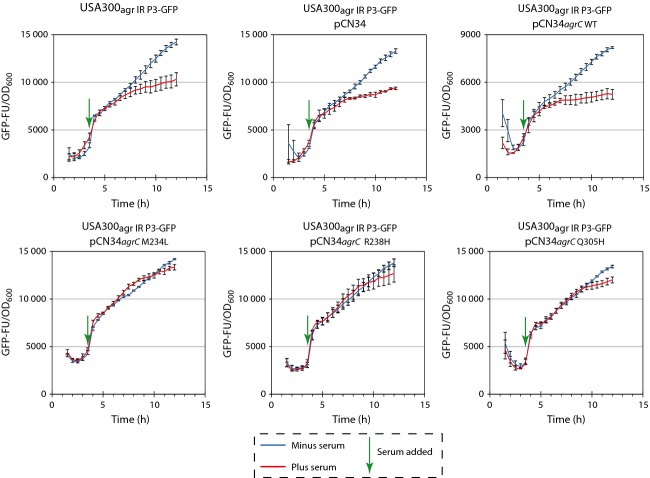
Graphs showing GFP expression [as GFP fluorescence units (GFP-FU)] as a function of growth (OD_600_) over time for *Staphylococcus aureus* USA300_agr_ _IR__P3-__GFP_ strain containing plasmids encoding for mutant and wild-type forms of AgrC (as indicated) grown in TSB with 25% (v/v) human serum added after cells reached late-exponential growth phase (indicated with a green arrow). Data shown were obtained from three biological replicates.

Having shown that intracellular levels of AgrA remain unchanged during growth in human serum, we investigated whether AgrA was subjected to any post-translational modifications that render it inactive for the activation of transcription from P2 and P3 promoters. We reasoned that if sequestration of AIP is the only mechanism by which the *agr* operon is downregulated during growth in human serum, then a constitutively active form of AgrC (that does not require AIP binding for autophosphorylation) should allow transcription from P2 and P3 to occur and thereby indicating that (i) AgrA is not subjected to any post-translational modification during growth in human serum that renders it inactive and (ii) transcription form P2 and P3 is not subject to an additional level of transcriptional regulation during growth in human serum through the action of other transcription regulatory proteins that affect transcription from P2 and P3. To experimentally test this, we introduced wild-type and previously identified constitutively active mutant forms of *argC* into our *S. aureus* USA300_agr IR P3-GFP_ reporter strain on a low copy number plasmid and under control of the P2 promoter (Geisinger *et al*., 2009). As the production of AgrC required AgrA to be produced (to activate P2), we first allowed AgrA levels to increase to a moderate level (approximately one-fifth of the maximal level) and then ‘induced’ the downregulation of *agr* expression by adding human serum to late-exponentially growing bacterial cells. As shown in Fig.4, GFP expression (indicating P3 transcription) was downregulated upon addition of human serum in the *S. aureus* USA300_agr IR P3-GFP_ reporter strain containing the empty plasmid or plasmid harbouring the wild-type *agrC*, whilst the cells containing plasmids encoding constitutively active forms of AgrC had no detectable downregulation of GFP expression upon addition of human serum. This result indicates that transcriptional downregulation of *agr* expression during growth in human serum is restricted to sequestration of the AIP by apolipoprotein B and thus is not subjected to regulation through transcription regulatory factors that either directly or indirectly affect transcription form P2 and P3.

## Discussion

Quorum-dependent regulation of virulence gene expression is a common strategy used by bacterial pathogens to ensure that the appropriate set of virulence genes are expressed at the correct time during infection. Thus, sequestration of the quorum-signalling molecule is an effective mechanism used by the host innate defence system to limit the quorum-dependent expression of bacterial virulence genes (Gordon *et al*., 2013). In the case of *S. aureus*, the *agr* operon mediates the quorum-dependent expression of the *S. aureus* virulon, and the level of *agr* operon expression is downregulated during growth in human serum, due to the sequestration of the *agr* quorum-signalling molecule by apolipoprotein B as part of the innate host defence mechanism. In this study, we have shown that transcriptional downregulation of *agr* expression during growth of *S. aureus* in human serum is restricted to sequestration of the AIP by apolipoprotein B and thus is not subjected to regulation through transcription regulatory factors that either directly or indirectly affect transcription form P2 and P3 (e.g. by transcription repressors that occlude the RNA polymerase from binding to P2 and P3 promoters or reduce the efficiency by which transcription initiates from P2 and P3). Importantly, we have shown that the transcriptional downregulation of *agr* expression in human serum can be overcome using constitutive active mutant forms of the quorum sensor AgrC, and therefore, it seems that AgrA is unlikely to be subjected to post-translational modifications that render it unavailable or inactive. Targeting bacterial quorum-sensing systems that are nonessential for growth but essential for virulence has been suggested as a novel strategy to curtail and manage bacterial infections, including those caused by *S. aureus*, *E. coli* and *Pseudomonas aeruginosa* (Gray *et al*., 2013; Melamed Yerushalmi *et al*., 2013). A study by Shopsin *et al*. revealed that the *agrAC* locus is a ‘hotspot’ for acquiring mutations in clinical isolates of *S. aureus* (Shopsin *et al*., 2010). Therefore, it is conceivable that mutations that confer constitutively active phenotypes could occur with increased frequency in the presence of an appropriate selection pressure (such as quorum-sensing inhibitors). Therefore, our results are relevant to the strategies that consider using quorum-sensing inhibitors since mutations that might occur in *agrC* or *agrA* through natural selection, and confer the constitutive active phenotype, could limit the effectiveness of such strategies in the long term. In addition, *as agr* dysfunction has been associated with persistent bacteraemia in MRSA (Chong *et al*., 2013), it is conceivable that downregulation of *agr* activity, as part of host, the innate immune response could in fact contribute to the formation of staphylococcal persisters leading to chronic infections.

## Supporting Information

Fig S1 Photograph of a bacterial growth plate (one of three biological replicates) taken on blue light transluminator to determine the stability of transcriptional reporters used in this study.

Fig S2 Western blot showing specificity of anti-AgrA polycolonal antibody to AgrA.

Table S1 Primers used for cloning, colony PCR, qRT-PCR and site-directed mutagenesis (SDM).

## References

[b1] Boles BR, Thoendel M, Roth AJ, Horswill AR (2010). Identification of genes involved in polysaccharide-independent *Staphylococcus aureus* biofilm formation. PLoS ONE.

[b2] Charpentier E, Anton AI, Barry P, Alfonso B, Fang Y, Novick RP (2004). Novel cassette-based shuttle vector system for gram-positive bacteria. Appl Environ Microbiol.

[b3] Cheung GY, Wang R, Khan BA, Sturdevant DE, Otto M (2011). Role of the accessory gene regulator agr in community-associated methicillin-resistant *Staphylococcus aureus* pathogenesis. Infect Immun.

[b4] Chong YP, Kim ES, Park SJ (2013). Accessory gene regulator (agr) dysfunction in *Staphylococcus aureus* bloodstream isolates from South Korean patients. Antimicrob Agents Chemother.

[b5] Dunman PM, Murphy E, Haney S (2001). Transcription profiling-based identification of *Staphylococcus aureus* genes regulated by the agr and/or sarA loci. J Bacteriol.

[b6] Fey PD, Endres JL, Yajjala VK, Widhelm TJ, Boissy RJ, Bose JL, Bayles KW (2013). A genetic resource for rapid and comprehensive phenotype screening of nonessential *Staphylococcus aureus* genes. MBio.

[b7] Geisinger E, Muir TW, Novick RP (2009). agr receptor mutants reveal distinct modes of inhibition by staphylococcal autoinducing peptides. P Natl Acad Sci USA.

[b8] Gordon CP, Williams P, Chan WC (2013). Attenuating *Staphylococcus aureus* virulence gene regulation: a medicinal chemistry perspective. J Med Chem.

[b9] Gray B, Hall P, Gresham H (2013). Targeting agr- and agr-like quorum sensing systems for development of common therapeutics to treat multiple gram-positive bacterial infections. Sensors (Basel).

[b10] Hall PR, Elmore BO, Spang CH (2013). Nox2 modification of LDL is essential for optimal apolipoprotein B-mediated control of agr type III *Staphylococcus aureus* quorum-sensing. PLoS Pathog.

[b11] Horsburgh MJ, Aish JL, White IJ, Shaw L, Lithgow JK, Foster SJ (2002). sigmaB modulates virulence determinant expression and stress resistance: characterization of a functional rsbU strain derived from *Staphylococcus aureus* 8325-4. J Bacteriol.

[b12] Kaito C, Saito Y, Ikuo M (2013). Mobile genetic element SCC-encoded RNA suppresses translation of and attenuates MRSA virulence. PLoS Pathog.

[b13] Klevens RM, Morrison MA, Nadle J (2007). Invasive methicillin-resistant *Staphylococcus aureus* infections in the United States. JAMA.

[b14] Koenig RL, Ray JL, Maleki SJ, Smeltzer MS, Hurlburt BK (2004). *Staphylococcus aureus* AgrA binding to the RNAIII-agr regulatory region. J Bacteriol.

[b15] Lee CY, Buranen SL, Ye ZH (1991). Construction of single-copy integration vectors for *Staphylococcus aureus*. Gene.

[b16] Malachowa N, Whitney AR, Kobayashi SD (2011). Global changes in *Staphylococcus aureus* gene expression in human blood. PLoS ONE.

[b17] Melamed Yerushalmi S, Buck ME, Lynn DM, Lemcoff NG, Meijler MM (2013). Multivalent alteration of quorum sensing in *Staphylococcus aureus*. Chem Commun (Camb).

[b18] Novick RP, Geisinger E (2008). Quorum sensing in staphylococci. Annu Rev Genet.

[b19] Peng HL, Novick RP, Kreiswirth B, Kornblum J, Schlievert P (1988). Cloning, characterization, and sequencing of an accessory gene regulator (agr) in *Staphylococcus aureus*. J Bacteriol.

[b20] Peterson MM, Mack JL, Hall PR (2008). Apolipoprotein B Is an innate barrier against invasive *Staphylococcus aureus* infection. Cell Host Microbe.

[b21] Reyes D, Andrey DO, Monod A, Kelley WL, Zhang G, Cheung AL (2011). Coordinated regulation by AgrA, SarA, and SarR to control agr expression in *Staphylococcus aureus*. J Bacteriol.

[b22] Reynolds J, Wigneshweraraj S (2011). Molecular insights into the control of transcription initiation at the *Staphylococcus aureus* agr operon. J Mol Biol.

[b23] Rothfork JM, Timmins GS, Harris MN (2004). Inactivation of a bacterial virulence pheromone by phagocyte-derived oxidants: new role for the NADPH oxidase in host defense. P Natl Acad Sci USA.

[b24] Shadrin A, Sheppard C, Severinov K, Matthews S, Wigneshweraraj S (2012). Substitutions in the *Escherichia coli* RNA polymerase inhibitor T7 Gp2 that allow inhibition of transcription when the primary interaction interface between Gp2 and RNA polymerase becomes compromised. Microbiology.

[b25] Shopsin B, Eaton C, Wasserman GA (2010). Mutations in agr do not persist in natural populations of methicillin-resistant *Staphylococcus aureus*. J Infect Dis.

[b26] Stock AM, Robinson VL, Goudreau PN (2000). Two-component signal transduction. Annu Rev Biochem.

[b27] Sun F, Liang H, Kong X (2012). Quorum-sensing agr mediates bacterial oxidation response via an intramolecular disulfide redox switch in the response regulator AgrA. P Natl Acad Sci USA.

[b28] Traber KE, Lee E, Benson S, Corrigan R, Cantera M, Shopsin B, Novick RP (2008). agr function in clinical *Staphylococcus aureus* isolates. Microbiology.

[b29] West AH, Stock AM (2001). Histidine kinases and response regulator proteins in two-component signaling systems. Trends Biochem Sci.

[b30] Wuster A, Babu MM (2008). Conservation and evolutionary dynamics of the agr cell-to-cell communication system across firmicutes. J Bacteriol.

