# Supplementary material for: Transcriptional downregulation of agr expression in Staphylococcus aureus during growth in human serum can be overcome by constitutively active mutant forms of the sensor kinase AgrC
Source: FEMS Microbiol Lett. 2013 Nov 18;349(2):153–62. doi: 10.1111/1574-6968.12309 (PMC4274972; doi:10.1111/1574-6968.12309)
Supplement: Data S1 — Materials and methods. [file fml0349-0153-SD1.docx]

| Primer | Use | Sequence |
| --- | --- | --- |
| P3 F (for GFP) | Cloning P3-GFP into pCL55;  fuse to GFP. *EcoR*I site. | CCGGAATTCTATATTGCCTAACTGTAGG |
| P3 R (for GFP) | Cloning P3-GFP into pCL55;  fuse to GFP. | ACATCTCTGTGATCTAGTTATATTAAAACATGC |
| P2 F (for GFP) | Cloning P2-GFP into pCL55;  fuse to GFP. *EcoR*I site. | CCGGAATTCTTTTTCTTAACTAGTCG |
| P2 R (for GFP) | Cloning P2-GFP into pCL55;  fuse to GFP. | ACCACTCTCCTCACTGTCATTATACG |
| P2 F (for *agrC*) | Cloning P2-*agrC* into pCN34;  fuse to *agrC*. *BamH*I site. | CGCGGATCCCCGGAATTCTTTTTCTTAACTAGTCG |
| P2 R (for *agrC*) | Cloning P2-*agrC* into pCN34;  fuse to *agrC*. | GGTATTGTAAACATTAATATCATTTTTTTTCCTCCTTAGGATCACC |
| GFP F (for P3) | Cloning P3-GFP into pCL55;  fuse to P3. Contains RBS. | CTAGATCACAGAGATGTGATCCTAAGGAGG |
| GFP F (for P2) | Cloning P2-GPF into pCL55;  fuse to P2. Contains RBS. | CAGTGAGGAGAGTGGTGATCCTAAGGAGG |
| GFP R | Cloning P3-GFP and P2-GPF into pCL55; fuse to P3 or P2. *BamH*I site. | CGCGGATCCTTATTTGTATAGTTCATCCATGCC |
| *agrC* F | Cloning P2-*agrC* into pCN34;  fuse to P2. | ATGATATTAATGTTTACAATACCAGC |
| *agrC* R | Cloning P2-*agrC* into pCN34;  fuse to P2. *Kpn*I site. | CGGGGTACCCTAGTTGTTAATAATTTCAAC |
| *agrC* M234L F | SDM of pCN34*_agrC_* _WT_ to M234L | GCTATCAACAACGAATTGCGCAAGTTCCGTCATG |
| *agrC* M234L R | SDM of pCN34*_agrC_* _WT_ to M234L | CATGACGGAACTTGCGCAATTCGTTGTTGATAGC |
| *agrC* R238H F | SDM of pCN34*_agrC_* _WT_ to R238H | CGAAATGCGCAAGTTCCATCATGATTATGTCAATATC |
| *agrC* R238H R | SDM of pCN34*_agrC_* _WT_ to R238H | GATATTGACATAATCATGATGGAACTTGCGCATTTCG |
| *agrC* Q305H F | SDM of pCN34*_agrC_* _WT_ to Q305H | CGAAAATTTTACGTGCACACGAAATGAATATTCCG |
| *agrC* Q305H R | SDM of pCN34*_agrC_* _WT_ to Q305H | CGGAATATTCATTTCGTGTGCACGTAAAATTTTCG |
| *geh* F | Integration test primer | GTTGTTTTTGTACATGGATTTTTAG |
| *geh* R | Integration test primer | CTTGCTTTCAATTGTGTTCC |
| pCL55 R | Integration test primer | GCGCATAGGTGAGTTATTAGC |
| *hld* F | qRT-PCR | GTGAATTTGTTCACTGTGCG |
| *hld* R | qRT-PCR | GGAAGGAGTGATTTCAATGG |
| *hld* probe | qRT-PCR | GTGACCGATTGTTGAAATG |
| *agrA* F | qRT-PCR | CTACAAAGTTGCAGCGATGG |
| *agrA* R | qRT-PCR | GCGTGTATGTGCAGTTTC |
| *agrA* probe | qRT-PCR | CCAGCTGAATTAAGAACTCG |
| *gyrB* F | qRT-PCR | CCTCAATTCGAAGGTCAAACGA |
| *gyrB* R | qRT-PCR | TCGTTCAAAGTGCTCTGAGAA |
| *gyrB* probe | qRT-PCR | TCTGAAGTGCGTCAAGTTGT |

**Supporting Information**

**Supplementary Table 1.** Primers used for cloning, colony PCR, qRT-PCR and site-directed mutagenesis (SDM).

**Supplementary materials and methods**

**Bacterial strains, plasmids and DNA manipulation**

Where appropriate, antibiotics were added to the growth media at the following concentrations: ampicillin, 100 μg/ml (*E. coli*); kanamycin, 90 μg/ml (*S. aureus*); and chloramphenicol, 7.5 μg/ml (*S. aureus*). Plasmid DNA was introduced into *E. coli* XL1-blue cells by heat-shock transformation. Plasmid DNA was introduced *S. aureus* RN4220 by electroporation with a Gene Pulser (Bio-Rad) with settings: 2.5 kV, 0.25 μFD, 100 Ω and in a 1 mm cuvette. Following this, cells were recovered in brain-heart infusion (BHI) supplemented with 0.5 M sucrose for 1 hour before being plated onto the appropriate selection plates. Transduction into target strains of *S. aureus* was carried out with φ11 following published protocols ([McNamara, 2008](#_ENREF_7)). All restriction enzymes and DNA modification enzymes were purchased from New England BioLabs. Plasmids and genomic DNA were extracted with the Qiagen plasmid miniprep kit and the Wizard bacterial genomic DNA kit (Promega), respectively, according to the manufacturers' instructions.

**Construction of *S. aureus* USA300 strain transcription reporters for P2 and P3**

The P2 and P3 promoters of the *agr* operon together with the complete intergenic region (*agr* intergenic region, IR, defined here as the sequence between the consensus -35 elements of P2 and P3 taken from the genome sequence of *S. aureus* subsp. aureus NCTC 8325 (GenBank accession 007795) ([Koenig*, et al.*, 2004](#_ENREF_5)) were PCR amplified from *S. aureus* SH1000 genomic DNA using primers P3 F, P3 R, P2 F and P2 R (for GFP; Supplementary Table 1). An RBS was included in the forward primers GFP F (for P2 and P3) used to amplify the GFPmut2 gene ([Cormack*, et al.*, 1996](#_ENREF_2)). The reverse primers (P2 and P3 R for GFP) of the promoter fragments contained a region homologous to the RBS. The two fragments (promoter and GFP) were fused together by PCR with primers (P2 F and GFP R (for P2) and P3 F and GFP R (for P3)) containing restriction sites *EcoR*I and *BamH*I on 5’ and 3’ ends respectively. The promoter fusion fragment was then ligated into the single-site integration vector pCL55 ([Lee*, et al.*, 1991](#_ENREF_6)) and transformed into *E. coli* XL1-blue cells and selected for using ampicillin, isolated and introduced into *S. aureus* RN4220 by electroporation to allow integration into the *S. aureus* genome at the *geh* locus *att_B_* site. Phage φ11 lysates were generated from the RN4220 strains were then transduced into the relevant destination strains by transduction and selected for using chloramphenicol. Following successful transduction, colonies were tested for integration of pCL55 into the *geh* locus using two primer pairs; one gives a PCR product if integration has not occurred (*geh* F and *geh* R; product approximately 1 kb) the other gives a PCR product if integration has occurred (*geh* F and pCL55 R; product approximately 1.5 kb).

**Bacterial growth and GFP expression assays**

Seed cultures were grown at 37°C, shaking at 700 r.p.m. for 6–7 h in a THERMOstar (BMG Labtech) plate incubator by directly inoculating a colony from a fresh LB agar plate into 200 µl of TSB medium in a 96-well microtitre plate (Sterilin). Following this, the seed cultures were diluted 1:50 in a final volume of 200 µl of fresh TSB medium (or 150 μl TSB for human serum “induction” assays) or the relevant ratios of TSB and human serum (5-50% (v/v) final concentration) to a final volume of 200 μl for human serum titration assay. Plates were incubated at 37°C, shaking at 500 r.p.m. For experiments shown in Fig. 2b, 2c, 3 and 4, 25% (v/v) human serum or fresh TSB was added to cells in the late exponential phase (as indicated in the Fig. 2b, 2c and 4). Data points for all experiments were gathered every 30 min unless otherwise indicated. GFP fluorescence expression data is presented as GFP fluorescence units (GFP-FU) relative to OD_600_ (GFP-FU/OD_600_).

**P2-AgrC plasmid construction**

The P2 promoter region (as described above) was amplified from the pCL55-P2GFP plasmid containing a region at the 3’ end homologous to the 5’ end of the *agrC* gene of *S. aureus* SH1000 strain and including the RBS as above (primers P2 F and P2 R for *agrC*). The *agrC* gene was then amplified from SH1000 genomic DNA (primers *agrC* F and *agrC* R) and the two fragments fused together by PCR using primers containing restriction sites *Bam*HI and *Kpn*I at the 5’ and 3’ ends respectively. The fragment was ligated into the low copy number plasmid pCN34 ([Charpentier*, et al.*, 2004](#_ENREF_1)) to create pCN34-*agrC* WT. Constitutively active mutant forms of *agrC* (M234L, R238H and Q305H ([Geisinger*, et al.*, 2009](#_ENREF_3))) were constructed using the QuikChange Site-Directed PCR Mutagenesis kit (Stratagene) using pCN34-*agrC* WT as a template and verified by sequencing. The pCN34-*agrC* WT and mutant plasmids were then introduced into USA300_WT_ by the following method: DNA was obtained from *E. coli* (screened for resistance to ampicillin) and introduced into *S. aureus* RN4220 by electroporation. Plasmid DNA was then recovered from RN4220 and subsequently introduced into the relevant destination strain by electroporation and positive transformants were selected on a LB agar plate containing kanamycin.

**RNA extraction and cDNA synthesis**

Samples (0.5 ml) were taken from 16 hr cultures of *S. aureus* USA300_WT_ and USA300_agr IR P3-GFP_. These were treated with lysostaphin (0.1 mg/ml) for 20 min and subsequently with 0.05% SDS solution for 5 min. Following this they were incubated with RNA stabilising agent TRIzol (Life Technologies) for 5 min. Total RNA was extracted using a Qiagen RNAeasy extraction kit and cDNA was synthesised from 1.5 μg RNA using a Qiagen omniscript reverse-transcription kit with random hexanucleotides (Promega); both according to manufacturer’s instructions.

**Western blotting to determine intracellular levels of AgrA**

For the experiment shown in Fig. 3, the bacterial cultures were grown as described above except that the cells were grown in 3 ml TSB in universal culture tubes. Cells were sampled at T_0_ (immediately before addition of human serum) and 2 and 16 hours (T_2_ and T_16_, respectively) after adding 25% (v/v) human serum to exponentially growing bacterial cultures (making the total volume 4 ml). Where indicated, 10 μg/ml of tetracycline was added together with the human serum to prevent *de novo* translation of AgrA mRNA (see Fig. 3).   For the experiment shown in Fig. S2, bacterial cultures were grown in 5 ml TSB in universal culture tubes. Samples were taken after 16 hr. Cell pellets were resuspended in 200 μl TSB and an equal number of cells (determined by measurement of OD 600 nm) were then pelleted and resuspended in 25 μl TSM buffer (50 mM Tris-HCl pH 7.5; 0.5 M sucrose and 10 mM MgCl_2_) with 0.1 mg/ml lysostaphin and incubated at 37°C for 30 minutes. Equal volume of SDS-PAGE sample buffer was added to the sample volume and boiled for 5 minutes. Twenty microliters of each samples was loaded onto a 12.5% (w/v) denaturing SDS-PAGE gel and proteins separated at 200 V for approximately 50 minutes. A positive control sample of heart muscle kinase (HMK) tagged recombinant AgrA was included on each gel ([Reynolds & Wigneshweraraj, 2011](#_ENREF_8)). MagicMark XP Western Protein Standard (Life Technologies) was used as a molecular weight size marker.

**Supplementary Results**


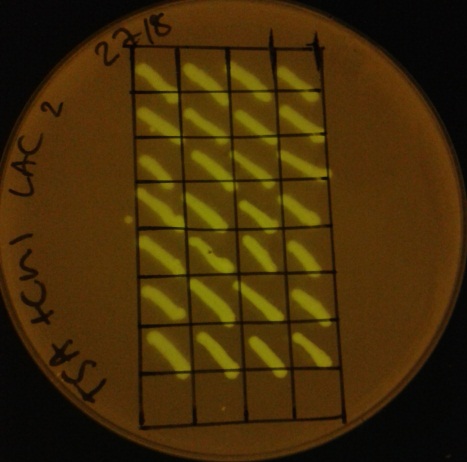


Fig. S1. Photograph of a bacterial growth plate (one of three biological replicates) taken on blue light transluminator to determine the stability of transcriptional reporters used in this study. Here, USA300_agr IR P3-GFP_ were cultured on TSA in the absence of antibiotic and 28 colonies from each plate (n = 3) examined for chloramphenicol resistance on TSA containing chloramphenicol. In each colony, chloramphenicol resistance was maintained and all colonies emitted green fluorescence when exposed to blue light indicating stable maintenance of the reporter.


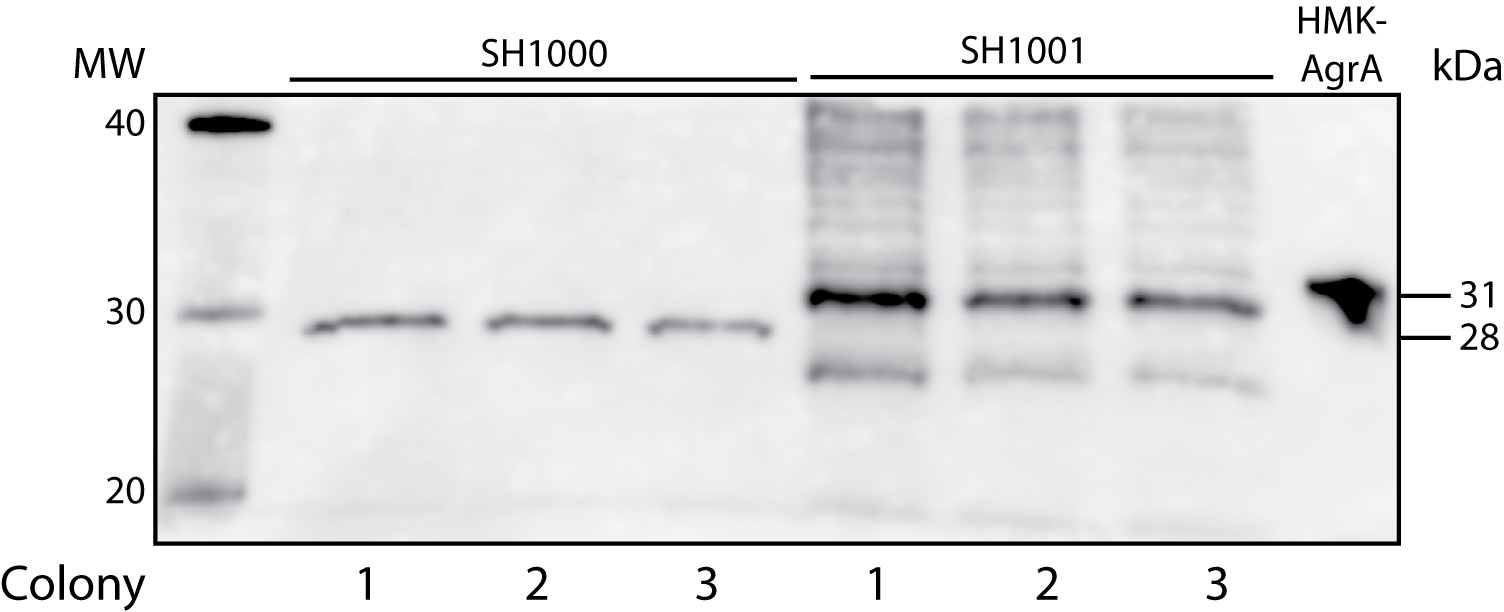


Fig. S2. Western blot demonstrating that the polyclonal anti-AgrA antibody generated and used in this study specifically recognises AgrA in whole-cell extracts prepared from three different colonies of *S. aureus* strain SH1000. As expected, the AgrA specific signal is not detectable in AgrA-deficient mutant *S. aureus* strain SH1001 ([Horsburgh*, et al.*, 2002](#_ENREF_4)).

Charpentier E, Anton AI, Barry P, Alfonso B, Fang Y & Novick RP (2004) Novel cassette-based shuttle vector system for gram-positive bacteria. *Appl Environ Microbiol* 70: 6076-6085.

Cormack BP, Valdivia RH & Falkow S (1996) FACS-optimized mutants of the green fluorescent protein (GFP). *Gene* 173: 33-38.

Geisinger E, Muir TW & Novick RP (2009) agr receptor mutants reveal distinct modes of inhibition by staphylococcal autoinducing peptides. *Proc Natl Acad Sci U S A* 106: 1216-1221.

Horsburgh MJ, Aish JL, White IJ, Shaw L, Lithgow JK & Foster SJ (2002) sigmaB modulates virulence determinant expression and stress resistance: characterization of a functional rsbU strain derived from Staphylococcus aureus 8325-4. *J Bacteriol* 184: 5457-5467.

Koenig RL, Ray JL, Maleki SJ, Smeltzer MS & Hurlburt BK (2004) Staphylococcus aureus AgrA binding to the RNAIII-agr regulatory region. *J Bacteriol* 186: 7549-7555.

Lee CY, Buranen SL & Ye ZH (1991) Construction of single-copy integration vectors for Staphylococcus aureus. *Gene* 103: 101-105.

McNamara PJ (2008) *Genetic manipulation of Staphylococcus aureus*. Caister Academic Press, 32 Hewitts Lane, Wymondham Nr 18 0ja, Uk.

Reynolds J & Wigneshweraraj S (2011) Molecular insights into the control of transcription initiation at the Staphylococcus aureus agr operon. *J Mol Biol* 412: 862-881.
